# Measuring Roadway Lane Widths Using Connected Vehicle Sensor Data

**DOI:** 10.3390/s22197187

**Published:** 2022-09-22

**Authors:** Justin A. Mahlberg, Howell Li, Yi-Ting Cheng, Ayman Habib, Darcy M. Bullock

**Affiliations:** Joint Transportation Research Program, Purdue University, West Lafayette, IN 47907, USA

**Keywords:** lane width, road maintenance, advanced driver assistance systems, ADAS, connected and autonomous vehicles, construction, camera detection

## Abstract

The United States has over three trillion vehicle miles of travel annually on over four million miles of public roadways, which require regular maintenance. To maintain and improve these facilities, agencies often temporarily close lanes, reconfigure lane geometry, or completely close the road depending on the scope of the construction project. Lane widths of less than 11 feet in construction zones can impact highway capacity and crash rates. Crash data can be used to identify locations where the road geometry could be improved. However, this is a manual process that does not scale well. This paper describes findings for using data from onboard sensors in production vehicles for measuring lane widths. Over 200 miles of roadway on US-52, US-41, and I-65 in Indiana were measured using vehicle sensor data and compared with mobile LiDAR point clouds as ground truth and had a root mean square error of approximately 0.24 feet. The novelty of these results is that vehicle sensors can identify when work zones use lane widths substantially narrower than the 11 foot standard at a network level and can be used to aid in the inspection and verification of construction specification conformity. This information would contribute to the construction inspection performed by agencies in a safer, more efficient way.

## 1. Introduction

The United States (US) has over three trillion vehicle miles of travel on over four million miles of public roadways, which require regular maintenance [1]. To maintain and improve these facilities, agencies often temporarily close lanes, reconfigure lane geometry, or completely close the road depending on the scope of the project. Areas leading to, through, and after the modified road configuration are all parts of the construction work zone. In the US, one work zone fatality occurs for every four billion vehicle miles of travel [2]. One of the largest issues with construction work zones is that it is often difficult to assess pavement markings, lane geometry and widths, and shoulder widths through work zones.

## 2. Background

**Figure 1:**
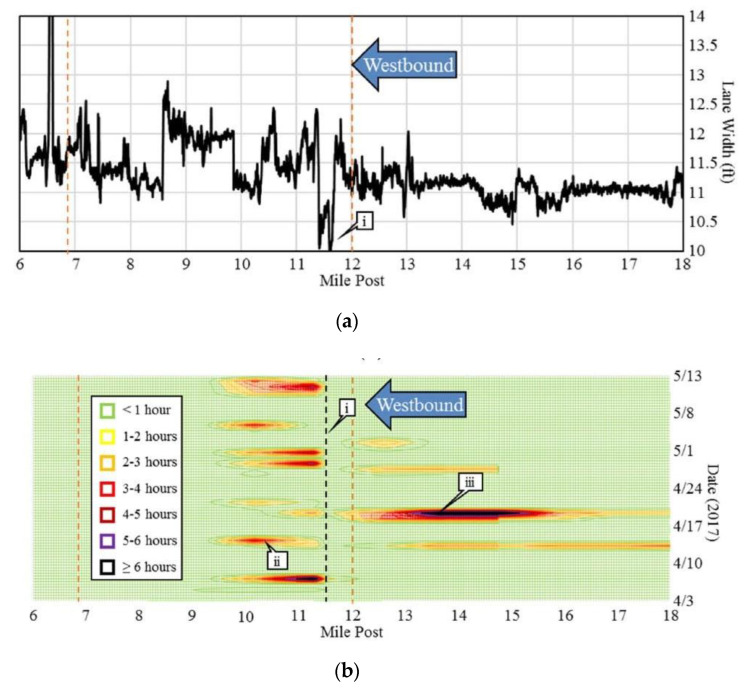
I-70 Westbound lane width and traffic speed changes; (**a**) longitudinal plot of lane width on I-70 in the westbound direction; and (**b**) frequency of speed changes greater than 15 miles per hour. Image source: Mekker, M. M., Y. J. Lin, M. K. I. Elbahnasawy, T. S. A. Shamseldin, H. Li, A.F. Habib, and D. M. Bullock. Application of LiDAR and Connected Vehicle Data to Evaluate the Impact of Work Zone Geometry on Freeway Traffic Operations. *Transportation Research Record*, Vol. 2672, No. 16, 2018, pp. 1–13. https://doi.org/10.1177/0361198118758050 (accessed on 10 April 2022) [6].

Previous studies have shown traffic volume, lane width, outside shoulder width, and pavement condition are contributing factors to crashes and have a direct impact on speed and capacity along US roads [3,4,5]. Another study captured geometric features through a work zone using LiDAR data and compared the impact of the collected features with vehicle speed; the key finding from that study can be seen in Figure 1 below. The graphic shows the lane widths through a work zone on I-70 in Indiana, which had a pronounced impact on the average travel speeds in the 2017 work zone [6]. It can be observed that when the lane widths are less than 11 feet (callout i), there is a substantial change in vehicle speeds and the resulting queues can lead to crashes [7].

Figure 1a shows linear referenced lane widths from mile marker 18 to mile marker 6 in the right lane of I-70 in the westbound direction. The work zone section is the area between the two dotted orange lines. There is a section on the roadway where the lane width decreases to 10 feet at mile marker 11.6 (callout i), while the INDOT minimum lane width allowed for this project is 11 feet. Additionally, near this location, the distance from the pavement markings to the barrier is less than 1 foot [6].

Figure 1b shows a spatiotemporal heat map of speed differences, or speed deltas, using probe vehicle speed data on I-70 westbound. The narrow section is indicated with the black dotted line. The speed delta between two adjacent segments is the difference in average speeds. Due to noise in smaller changes in speed, deltas greater than 15 mph were used. The color of the heatmaps shows the duration for which a given speed delta occurred. For example, a green speed delta indicates that a difference in speeds with adjacent segments greater than 15 mph occurred for less than one hour at that location and time. At mile marker 14 on April 19, the duration of the speed delta greater than 15 mph is more than 6 h [6].

In Figure 1a,b, callout i indicates the location where lane widths fall below 10 feet. In Figure 1b, callout ii corresponds to the congestion in the work zone shown. The heatmap shows recurring speed deltas as traffic approaches the narrow lane width. On 19 April 2017, (callout iii), there is a lane closure having a greater impact on traffic. Although the narrow section is short, this shows the lasting impact it has on traffic. Traffic queues extend beyond the work zone warning area. This is crucial as a previous study found that non-recurring mile-hours of congestion have a great influence on the crash rate. The congested crash rate on all Indiana interstates in 2014 was found to be 24 times greater than the uncongested crash rate [8].

## 3. Measuring Lane Widths with Connected Vehicle Data

The recent emergence of high-fidelity cameras equipped in modern production vehicles can detect the width of the vehicle driving lane using computer vision, machine learning, and measurement algorithms [9]. Conventionally, these computer vision and machine learning applications are used for Advanced Driver Assistance Systems (ADAS) to support level two autonomous driving, such as lane assist features. An opportunity has emerged to make use of this data to provide insight into geometric conditions on all roadways, but especially work zones where lane widths are often reduced. A drawback to the use of these sensors is the performance of the sensors in various weather conditions, including snow, fog, rain, and various geometric designs [10,11]. Although these conditions were not experienced during this study, the lack of readings can be eliminated in a crowdsourced data set, the benefit of using production vehicle data is that it can be crowdsourced and provide ubiquitous coverage across the state without resource-intensive deployments and field data collection efforts. This study explores the use of vehicle sensor data on rural and urban road networks for assessing the geometry of the driving lane, while camera and speed data are used for validation.

## 4. Motivation

**Figure 2:**
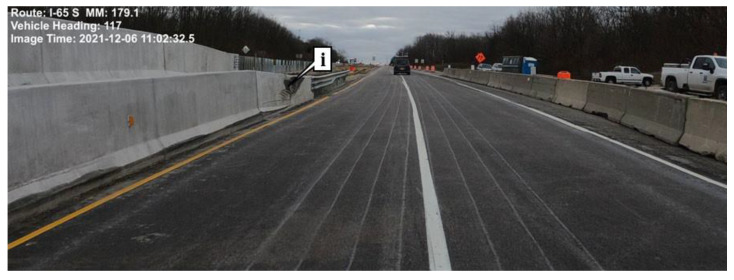
Capture of lane widths through the I-65 work zone at mile marker 179.

Studies have shown the importance of adequate lane widths for work zone safety [6,12]. Figure 2 shows a location on I-65 that experienced an increased crash rate during construction. Mobile LiDAR data was collected to capture lane widths and lateral clearance between the edge of the driving surface and the construction barrier. Figure 2 shows the southbound direction of I-65 at this location, and callout i shows a scuff mark on the construction barrier due to tire rubbing.

**Figure 3:**
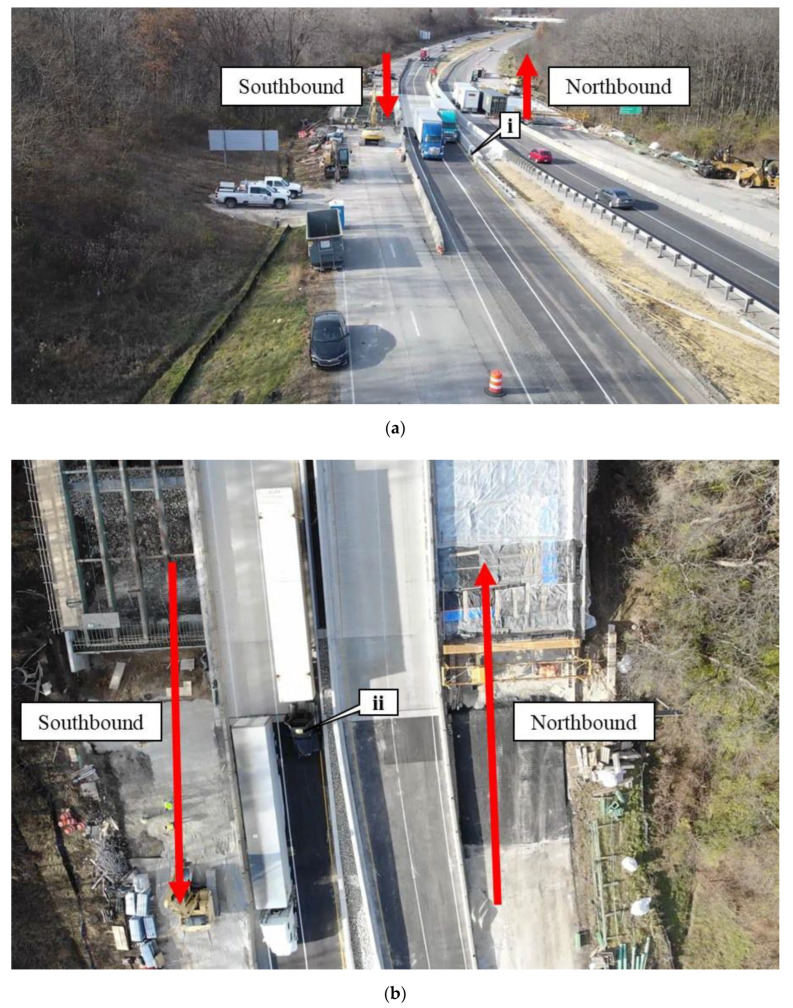
Aerial photos of the I-65 work zone at mile marker 179; (**a**) aerial view of I-65 work zone at mile marker 179; and (**b**) birds-eye view of I-65 work zone at mile 179.

Figure 3a shows an aerial view of the work zone and the respective travel directions. Callout i is the same scuff mark that is observed in Figure 2. In Figure 3a, as the two semis are traversing the work zone, there is not much tolerance for lateral deviation of the trucks. An image directly above the work zone can be seen in Figure 3b. Two semis are traveling through the work zone in the southbound lanes and the black semi (callout ii) is traveling over the left edge line close to the construction concrete barrier.

**Figure 4:**
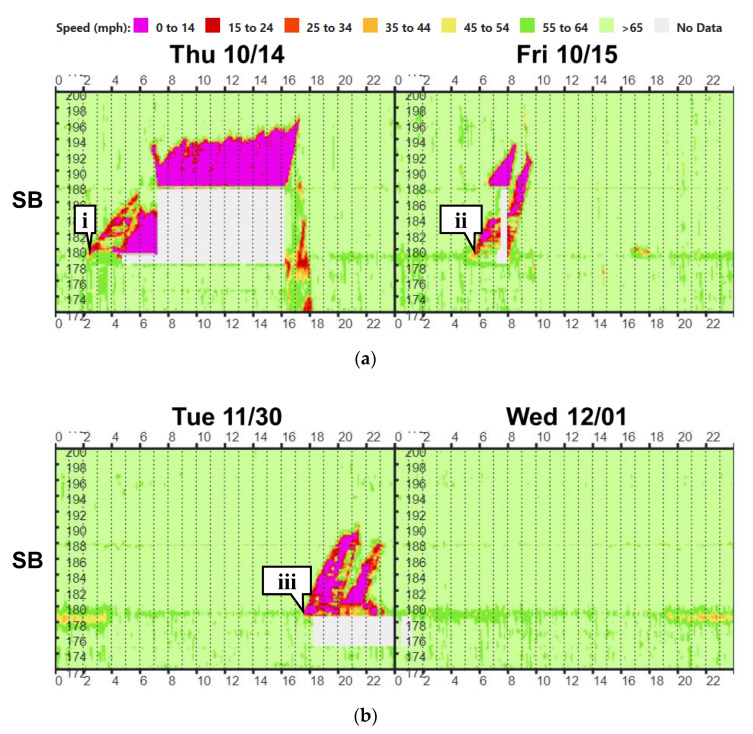
I-65 speed profile heatmap for three crashes and highway closures; (**a**) two crashes on 14–15 October 2021; and (**b**) one crash over 30 November with traffic impact lasting until 1 December 2021.

The spatiotemporal heatmap in Figure 4 shows vehicle speeds at this location by linear referenced 0.1 mile segments. The heatmaps have been extensively used in past research to visualize congested conditions [13,14,15,16]. The horizontal axis on the speed profile heatmaps represents the time of day while the vertical axis represents mile marker location. I-65 is shown in the southbound (SB) direction of travel from mile marker 200 to mile marker 172 in Figure 4. Figure 4a shows the heatmap from October 14 to October 15, 2021. Callout i corresponds to a semi-rollover. This crash caused the interstate to be shut down for almost 12 h, and the total impact on the southbound direction was almost 20 miles of interstate shutdown or slow-moving diverted traffic for a total recovery period of 16 h. Subsequent crashes at this location occurred on October 15 (callout ii) and November 30 (callout iii).

## 5. Objective

The objective of this study is to determine if lane width measurements from sensors onboard production vehicles can be used to screen public road networks for sections that have less than prescribed lane widths. Previous studies have found many uses for connected vehicle data, including pavement marking evaluation, traffic signal performance, and crash mitigation through surrogate safety measures and hard braking [17,18,19,20,21]. The benefit of using production vehicle data is that it can be crowdsourced and provide ubiquitous coverage across the state without resource-intensive deployments and data collection. The vehicle sensor data used in this study was validated through field measurements and mobile LiDAR data on rural and urban roadways.

### 5.1. Measuring Lane Widths with Mobile Mapping Units

**Figure 5:**
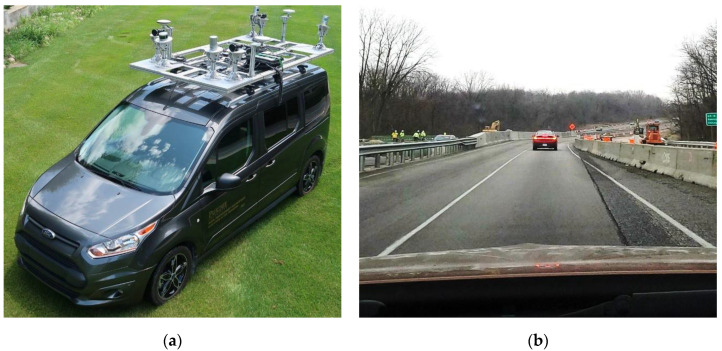
LiDAR and production vehicle data collection equipment; (**a**) Purdue Mobile Mapping System for LiDAR data collection; and (**b**) production vehicle for data collection.

Using a mobile LiDAR system, the data collection process on a 12 mile section of roadway took only 20 min once the system was set up and calibrated (Figure 5a). With the current data processing and reduction methods, the lane width measurements can be obtained within one day of the data collection. However, it is costly to deploy mobile LiDAR mapping every time a work zone is reconfigured or to perform studies over a large geographic area.

### 5.2. Measuring Lane Widths with Production Vehicles

Production vehicles instrumented with data loggers are utilized to collect lane width data for evaluation. The lane width value is a direct output provided from the vehicle that has been calculated through the ADAS camera located approximately 4 feet from the driving surface. The vehicle does the on-board edge computing from the camera images to determine the lane width. As a validation tool, a GoPro camera is mounted within the vehicle looking ahead to capture the roadway for validation. To validate the lane width measurements of the production vehicles, the Purdue Mobile Mapping System for LiDAR data collection is used to verify accuracy (Figure 5a). Production vehicle data is evaluated through a work zone on I-65 northbound at mile marker 179 (Figure 5b) and compared with the results from the mobile LiDAR platform.

## 6. Evaluation Protocol—Validation of Camera Detection Lane Widths Using LiDAR

**Figure 6:**
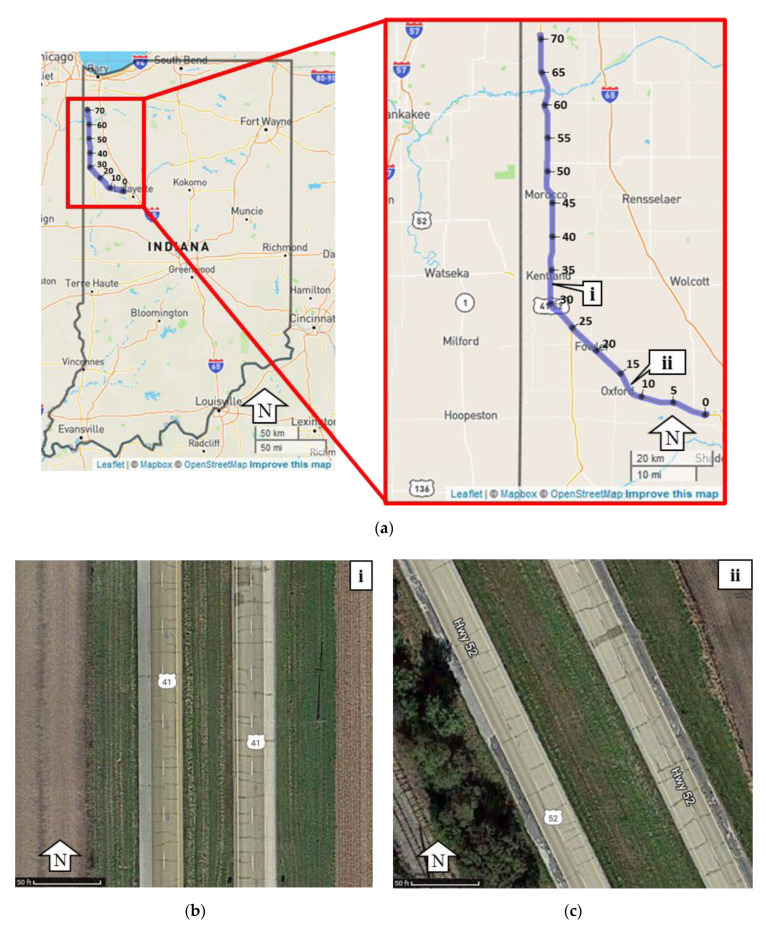
Data collection of LiDAR and production vehicle data on US-52 and US-41; (**a**) US-52 and US-41 data collection route; (**b**) US-41 typical cross section; and (**c**) US-52 typical cross section.

Ground-truth data are collected with GoPro cameras and mobile LiDAR on 14 July 2021, and 14 January 2022. The camera images are taken at half-second intervals, containing timestamps and GPS location in the metadata. Purdue’s Mobile Mapping System collects the LiDAR data for precise width validation by measuring the distance between the centerlines of the automatically derived lane markings from the acquired point clouds. The production vehicle provided 8831 measurements and the mobile mapping system provided 1,378,780 data points on the data collection route. The data collection routes are the US-52 and the US-41 from West Lafayette to Lowell, Indiana (Figure 6a). Figure 6b,c are satellite images of US-41/US-52 to provide context of a typical cross section along the data collection route. These locations correspond to callout i and callout ii, respectively in Figure 6a.

The Purdue Wheel-based Mobile Mapping System (PWMMS) is equipped with four LiDAR units: three Velodyne HDL-32Es units seen in the front left and rear of the vehicle, and one VLP-16 unit at the front right. The vehicle is also equipped with high-definition RGB cameras in the front left, front right, and rear. The LiDAR and imaging units are directly georeferenced with a global navigation satellite system/inertial navigation system (GNSS/INS) unit. Through a system calibration procedure, mounting parameters between camera/LiDAR units and a GNSS/Inertial Measurement Unit (IMU) navigation system are estimated, facilitating the reconstruction of georeferenced, well-registered/georeferenced point clouds from the LiDAR scanners [22]. There are many uses for the registered point clouds, including pavement marking evaluation, lane widths, pavement distress, and ditch line mapping [12,23,24,25]. For lane width estimation, the pavement markings are extracted from registered point clouds. The methods used to extract pavement markings follow the methodology proposed by Cheng et al. [22]. Once the pavement markings are identified, the marking centerline is derived and clustered to identify areas with ambiguous or missing lane markings before the lane width can finally be calculated [12].

**Figure 7:**
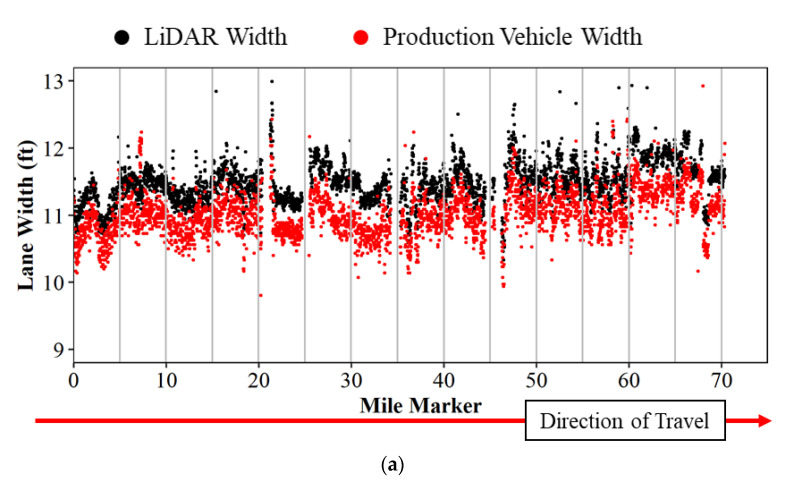

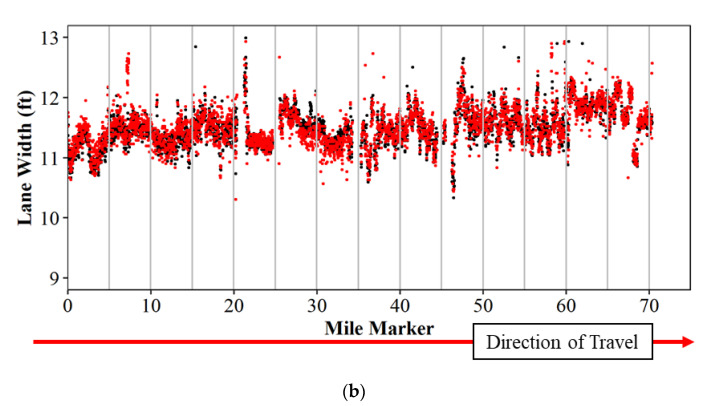
Lane width comparison of LiDAR and production vehicle data on US-41 and US-52 northbound; (**a**) no measurement offset; and (**b**) measurement offset of 6 inches.

Lane widths from mobile LiDAR and production vehicle data are compared by linear referencing each data point to the nearest 0.01 mile marker on the data collection route and by assigning the direction of travel using the vehicle heading. Once this is done, an average is taken for each 0.01 mile and plotted as a scatter plot in Figure 7a below. LiDAR lane widths are plotted in black, and production vehicle widths are plotted in red. The graph shows the mile marker along the horizontal axis and the lane width value observed on the vertical axis. There are a few anomalies observed that are caused by lane changes in the production vehicle and missing pavement markings through towns at mile marker 21. There is an approximate 6″ offset between the lane widths in Figure 7a because the LiDAR systems measured center to center of lines and the connected vehicle measures inside edge of line to inside edge of line. Figure 7b shows the same data, but with both measurements referenced from center of line to center of line.

**Figure 8:**
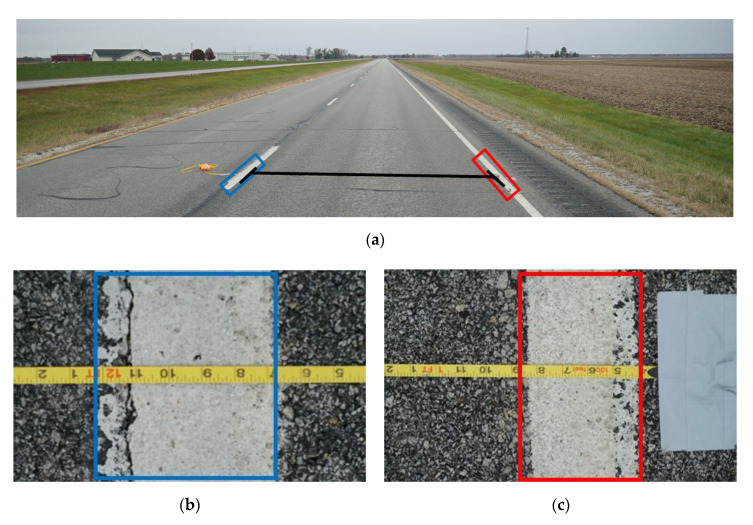

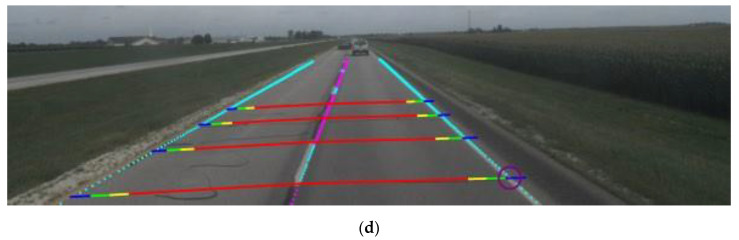
Field validation of lane widths; (**a**) field validation of lane widths with tape measure; (**b**) centerline view of tape measure; (**c**) edge line view of tape measure; and (**d**) field validation of lane widths with LiDAR data.

To provide a spot check on the data, the team performed field validation using manual measurement of lane widths on the study route with a tape measure (Figure 8a). The team measured from the inside edge of pavement marking to the opposite inside edge of pavement marking, midpoint of pavement marking to the midpoint of pavement marking, and the outside edge of pavement marking to the outside edge of pavement marking. An example of the measurements taken can be seen in Figure 8b for the centerline marking and Figure 8c for the right edge marking.

**Figure 9:**
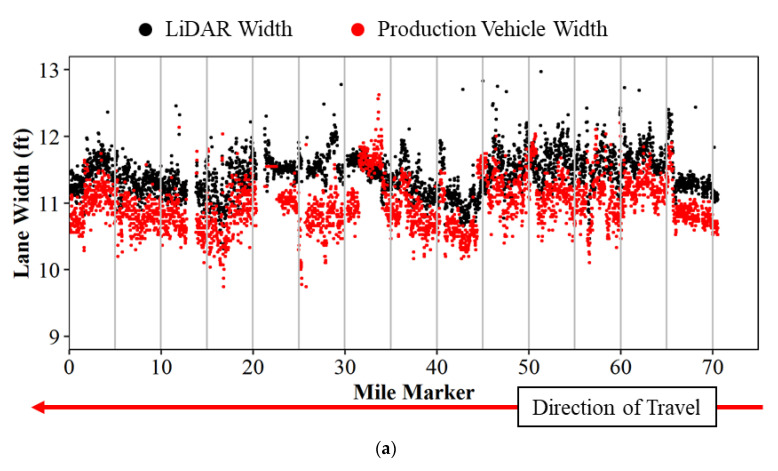

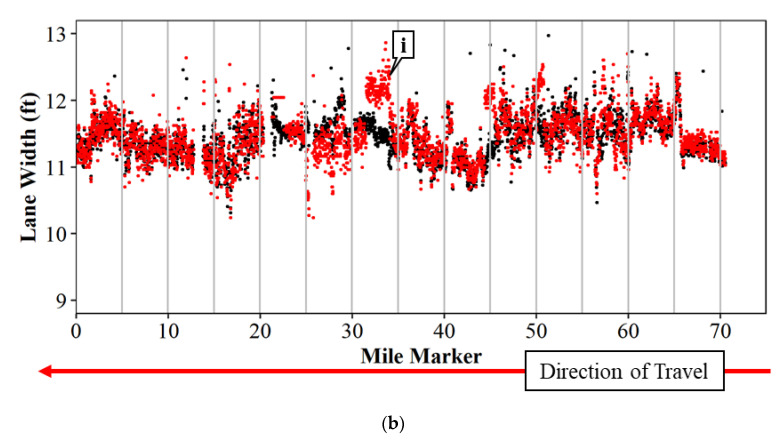
Lane width comparison of LiDAR and production vehicle data on US-41 and US-52 southbound; (**a**) no measurement offset; and (**b**) measurement offset of 6 inches.

The same analysis is performed in the southbound direction of the study route. Figure 9a shows the raw lane width for LiDAR width in black and production vehicle data width in red. Similar to the northbound direction, the 6 inch adjustment is needed to reconcile the difference in the measurement methods. Figure 9b shows the adjusted data. The trends in lane width remain consistent except for mile marker 33 (callout i). The GoPro images confirm that the production vehicle changed lanes at this location from the right lane to the left lane, while all mobile LiDAR-based lane width values are derived for the right lane.

## 7. Scalability

**Figure 10:**
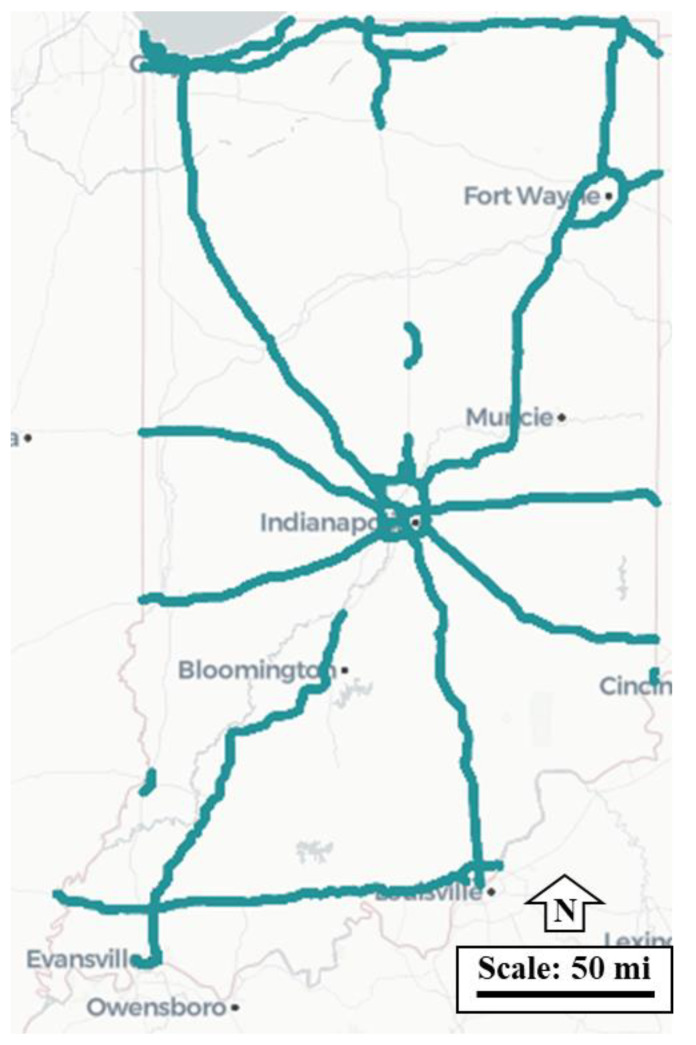
Crowdsourced data across Indiana.

Crowdsourced data from vehicles equipped with ADAS scales well for locating lane width challenges at a state level. A dataset of over 1.5 million data points generated from connected vehicles from 24 November 2021, to 29 March 2022, on limited-access roadways in the state of Indiana is shown in Figure 10.

This data is then linearly referenced to a corresponding route and mile post. The vehicle miles traveled for each interstate by direction are summarized in Table 1 below. For I-65 alone, there are over 160,000 data points traveling 8000 miles, with 581 vehicle trips taken. Redundancy is important to cover roadway sections with multiple lanes, lane change activity, environmental variables, and other traffic that may occlude line visibility. This data source provides an opportunity for agencies to assess their infrastructure quickly and efficiently.

**Figure 11:**
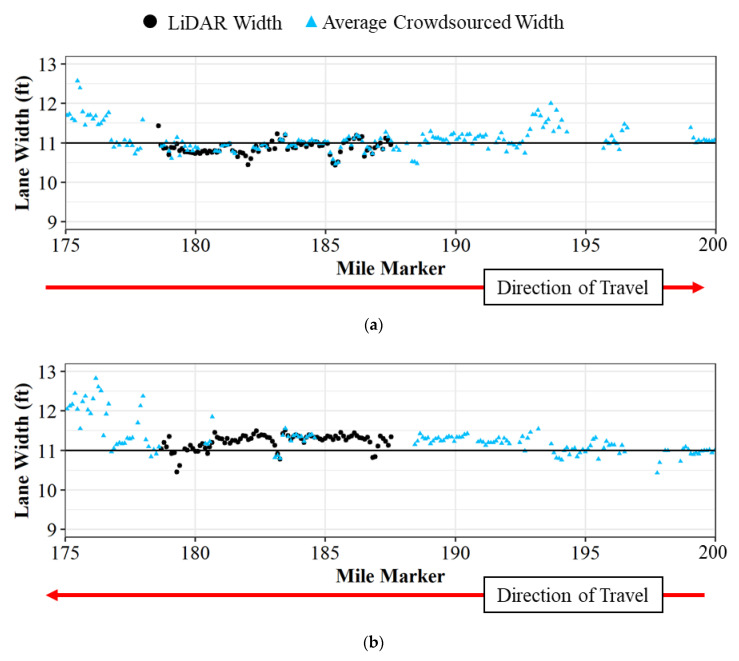
Lane width comparison on I-65 of LiDAR and crowdsourced lane width data; (**a**) northbound; and (**b**) southbound.

The crowdsourced production data is also validated with LiDAR-based lane widths. Figure 11 shows the average crowdsourced lane widths after the 6 inch adjustment and the average LiDAR widths on I-65 from mile marker 175 to mile marker 200. Figure 11a shows the lane widths on I-65 northbound and Figure 11b shows the lane widths on I-65 southbound. The lane widths from the crowdsourced data track well with the LiDAR width. The LiDAR widths are only provided from mile marker 178 to mile marker 188 as the Mobile Mapping System only collected data on that stretch of roadway, which also shows the limitation of single-vehicle data collection compared to crowdsourced data.

## 8. Identifying Lane Width Outliers with Connected Vehicle Data

**Figure 12:**
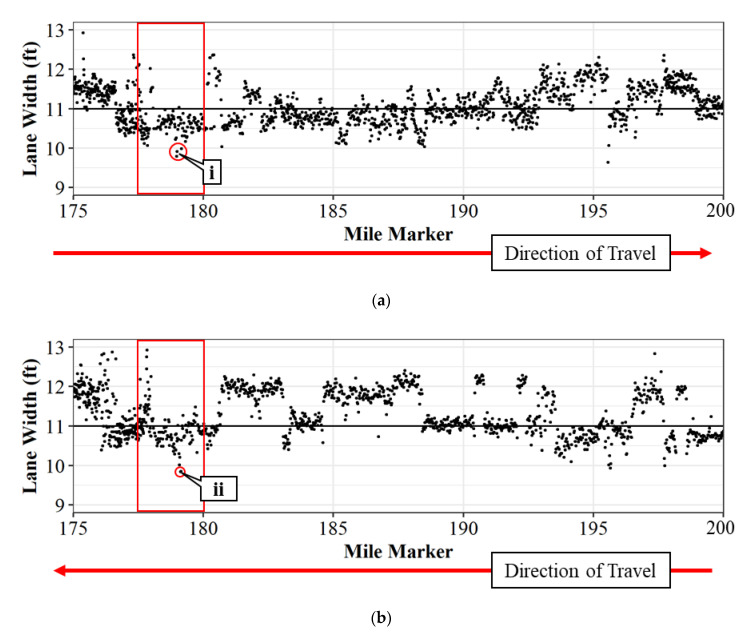
Lane widths using crowdsourced data for I-65 from mile marker 175 to 200; (**a**) northbound; and (**b**) southbound.

Crowdsourced data for 25 miles of interstate using three vehicles is shown in Figure 12. Figure 12a shows the lane widths in the northbound direction, and Figure 12b shows the lane widths in the southbound direction for only crowdsourced data. The red box shows the area of interest where the work zone resides. Callout i show locations through the work zone where lane widths fall below ten feet in the northbound direction, specifically at mile markers 178.97, 178.99, and 179.16. Callout ii pinpoints locations in the southbound direction where lane widths are also below ten feet, specifically at mile marker 179.12, which corresponds to the location identified in Figure 2. These locations were validated with the LiDAR widths and are also the places that have seen crashes over the construction period.

## 9. Conclusions and Recommendations

Traditional methods for evaluating lane widths by measuring with tapes and/or survey crews are impractical in many construction zones with limited work areas and high traffic volumes. The emergence of computer vision and machine learning technologies for driving ADAS functionality in production vehicles has presented a rich new data set for agencies to monitor construction zone lane widths. This study investigated whether lane width data from production vehicle sensors provides a sufficiently accurate and scalable data source for agencies to identify areas with narrow lane widths state-wide. The Purdue Wheel-based Mobile Mapping System with LiDAR units was used to evaluate the accuracy of the production vehicle data. Vehicle sensor data tracked closely with LiDAR-measured widths (Figure 7, Figure 9 and Figure 11) with a root mean square error of approximately 0.24 feet.

The novelty of these results is that onboard vehicle sensors can identify where work zones have lane widths substantially narrower than the 11 foot standard at a network level and can be used to aid in inspecting and validating construction work zones at scale. This information would contribute to the construction inspection performed by agencies in a safer, more efficient way. In the long term, it will be important to collect not only lane width but also lateral offset to obstructions such as barrier walls (Figure 2, callout i) and or guardrails.

## Figures and Tables

**Table 1:** Summary of crowdsourced data on Indiana Interstates.

Interstate (Total Miles)	Vehicle Miles Traveled (Total Unique Trips)					
	Northbound	Southbound	Eastbound	Westbound	Inner Loop	Outer Loop
I-265 (14)			355 (94)	353 (95)		
I-465 (106)					2822 (234)	2849 (294)
I-469 (62)	199 (14)	159 (17)				
I-64 (146)			862 (59)	854 (59)		
I-65 (524)	4194 (291)	3830 (290)				
I-69 (716)	3727 (225)	3560 (252)				
I-70 (314)			2208 (163)	1860 (144)		
I-74 (342)			905 (105)	947 (105)		
I-865 (10)			77 (60)	112 (50)		
I-94 (92)			1671 (109)	1239 (84)		

## Data Availability

Not applicable.
